# Effects of TiO_2_ Nanoparticle Doping on the Micro-Arc Oxidation Coating Structure and Corrosion Resistance of 6061 Aluminum Alloy

**DOI:** 10.3390/molecules31030468

**Published:** 2026-01-29

**Authors:** Zhu Huang, Shaodian Yang, Xiuxiang Liao, Shengxiang Yang, Tong Zhang, Bingchun Jiang

**Affiliations:** 1 College of Mechanical and Electrical Engineering, Guangdong University of Science and Technology, Dongguan 523000, China; hzhuangzhu@163.com (Z.H.); shaodianyang@163.com (S.Y.); xiuxiang_liao@foxmail.com (X.L.); 18785313440@163.com (S.Y.); 2 School of Mechanical and Electrical Engineering, Jiangxi University of Science and Technology, Ganzhou 341000, China; 3 School of Materials Science and Engineering, Hunan University of Science and Technology, Xiangtan 411201, China

**Keywords:** micro-arc oxidation, 6061 aluminum alloy, TiO_2_ nanoparticles, microstructure, corrosion resistance

## Abstract

To elucidate the effects of TiO_2_ nanoparticles on the microstructure and corrosion resistance of micro-arc oxidation (MAO) coatings formed on 6061 aluminum alloy, MAO coatings were prepared in a silicate-based electrolyte with varying TiO_2_ nanoparticle concentrations. The coating structure and properties were evaluated using a coating thickness gauge, surface profilometer, scanning electron microscopy (SEM), energy-dispersive X-ray spectroscopy (EDS), X-ray diffraction (XRD), X-ray photoelectron spectroscopy (XPS), and an electrochemical workstation. The results show that, with increasing TiO_2_ content, both coating thickness and surface roughness gradually increase, while the surface porosity first decreases and then increases. An appropriate amount of TiO_2_ effectively lowers the surface porosity and enhances coating compactness. The T_1_ condition exhibited the least precipitation of corrosion products during immersion tests and thus the best corrosion resistance. Compared to the untreated 6061 aluminum alloy substrate, the optimized coating demonstrated a reduction in corrosion current density (*J*_corr_) by more than one order of magnitude, reaching 1.127 × 10^−6^ A·cm^−2^, while its polarization resistance (*R*_p_) increased by over one order of magnitude, attaining 3.558 × 10^4^ Ω·cm^2^. Furthermore, relative to the TiO_2_-free T_0_ coating, the *J*_corr_ of the optimized coating was further reduced by approximately 2.5 times, with its *R*_p_ enhanced by about 2.3 times. XRD analysis indicated that the MAO coatings primarily consist of α-Al_2_O_3_ and γ- Al_2_O_3_. This study provides theoretical and experimental support for the application of TiO_2_ nanoparticles in MAO processes.

## 1. Introduction

Aluminum alloys, owing to their low density, excellent mechanical properties, favorable electrical and thermal conductivities, and outstanding formability, serve as core structural materials in automotive lightweighting, aerospace components, and electronic packaging [1,2,3,4]. However, their relatively low hardness, as well as inferior wear and corrosion resistance, limit their use in certain critical applications [5,6,7]. Toa overcome these limitations, various surface modification techniques have been developed, including mechanical treatment, laser surface processing, physical vapor deposition, chemical vapor deposition, anodizing, and micro-arc oxidation (MAO) [8,9,10,11,12,13]. MAO is widely employed to improve the performance and extend the service life of aluminum alloys due to its environmentally friendly, economical, and facile operation with low cost and minimal pollution. The core of MAO lies in forming a ceramic oxide layer firmly bonded to the substrate surface [14,15], thereby enhancing surface properties. Nevertheless, defects such as pores and microcracks generated during MAO can significantly reduce corrosion resistance and hinder further application [16,17].

Incorporating functional nanoparticles during MAO can increase coating density via pore-sealing effects, thereby improving performance [18,19]. For example, Zhang et al. [20] added MoS_2_ nanoparticles to a silicate electrolyte to fabricate wear-resistant and lubricious Al_2_O_3_/MoS_2_ nanocomposite coatings and found that MoS_2_ optimized the ceramic microstructure and improved wear resistance. Yu et al. [21] prepared black MAO ceramic coatings containing TiO_2_ nanoparticles on 6063 aluminum alloy, demonstrating that TiO_2_ increased coating thickness, reduced porosity, raised surface roughness, and markedly enhanced mechanical properties. Huang et al. [22] treated LY12 aluminum alloy in an electrolyte containing ZnO particles and found that ZnO suppressed coating damage and dissolution in high-salinity environments, significantly improving corrosion resistance.

Despite demonstrated potential, systematic process studies on the growth behavior and corrosion resistance of MAO coatings formed on 6061 aluminum alloy in silicate electrolytes with TiO_2_ nanoparticle addition remain limited. Moreover, prior analyses of coating microstructure often emphasize qualitative surface morphology, with relatively few reports quantitatively evaluating pore size and porosity.

Therefore, this study investigates the preparation of MAO coatings by doping TiO_2_ nanoparticles at varying concentrations in a silicate-based electrolyte, aiming to systematically explore the effect of doping levels on the structural and functional properties of the coatings. In this study, TiO_2_ nanoparticles were selected as the additive due to their superior multifunctional performance compared to traditional ceramic particles such as Al_2_O_3_ and SiO_2_ [23,24]. TiO_2_ nanoparticles not only act as a physical barrier, enhancing the compactness of the coating, but also, when doped at an appropriate concentration, contribute to increasing the oxidation voltage and intensifying the arc discharge effect. This promotes the formation of hard phases such as α-Al_2_O_3_ while suppressing the formation of large pores caused by excessive discharge. The selected TiO_2_ doping concentrations were determined based on preliminary feasibility tests and commonly reported effective doping ranges in the literature to ensure scientific rigor and comparability in the experimental design. In terms of performance expectations, the literature indicates that undoped MAO coatings on 6063 aluminum alloy typically exhibit relatively weak corrosion resistance in a 3.5% NaCl solution, with a corrosion current density of 3.44 × 10^−6^ A·cm^−2^ [25]. Building on this, the present work introduces TiO_2_ nanoparticles into the MAO process and employs image statistical methods to systematically quantify the surface pore size distribution of the coatings. By establishing correlations between pore size distribution, porosity, and electrochemical corrosion performance, this study aims to elucidate the regulatory mechanisms through which microstructure influences corrosion resistance. The findings are expected to provide both experimental evidence and theoretical insights for the development of high-performance MAO protective coatings.

## 2. Results

### 2.1. Evolution of Anodic Voltage

**Figure 1:**
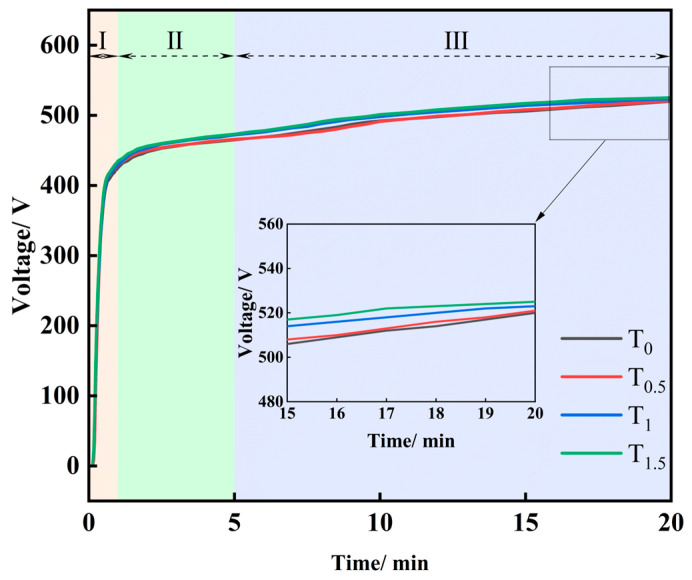
Anode voltage of MAO.

Figure 1 presents the anodic voltage versus time during MAO for different TiO_2_ loadings. All curves exhibit similar trends: the voltage rises with time, and a higher TiO_2_ concentration yields a higher final voltage. Three stages can be distinguished: anodizing, spark discharge, and micro-arc discharge. In the anodizing stage (first ~1 min), the voltage rapidly increases to ~400 V at ~400 V·min^−1^. In the spark discharge stage (1–5 min), the growth rate drops markedly to ~3% of that in the first stage. In the micro-arc stage (5–20 min), the voltage approaches a quasi-steady level with small fluctuations. As an electrical insulator, TiO_2_ decreases the electrolyte conductivity; increasing TiO_2_ loading raises the solution resistance and hinders ion transport. To maintain the set current, a higher voltage is required, leading to an increased final voltage.

### 2.2. Effects of TiO_2_ on Coating Thickness and Surface Roughness

**Figure 2:**
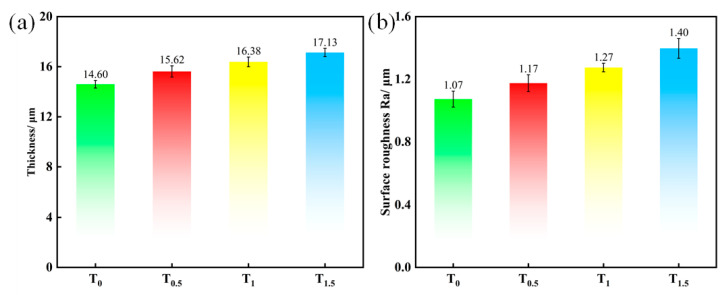
Coating thickness and surface roughness under different TiO_2_ nanoparticle doping contents: (**a**) thickness; (**b**) surface roughness.

Figure 2 shows that coating thickness increases monotonically with TiO_2_ concentration, indicating a positive correlation. The minimum and maximum thicknesses are 14.60 μm (T_0_) and 17.13 μm (T_1.5_), respectively (Figure 2a). This thickening is attributed to (i) the high surface activity of TiO_2_ nanoparticles, which form charged colloids by ion adsorption and migrate under the electric field to the growing surface to participate in and promote MAO reactions, and (ii) the insulating nature of TiO_2_, which raises the electrolyte resistance and local field strength, thereby increasing discharge frequency, generating more molten oxide, and depositing it onto the surface to further build thickness [26].

Surface roughness also increases with TiO_2_ loading (Figure 2b), from 1.07 μm (T_0_) to 1.40 μm (T_1.5_). Under the high-temperature/high-pressure micro-arc discharges, TiO_2_ nanoparticles can agglomerate and deposit on the coating surface; some agglomerates are embedded into the molten oxide layer, producing local protrusions, degrading surface flatness, and increasing roughness [27].

### 2.3. Effects of TiO_2_ on Surface Micro-Morphology

#### 2.3.1. Surface Morphology

**Figure 3:**
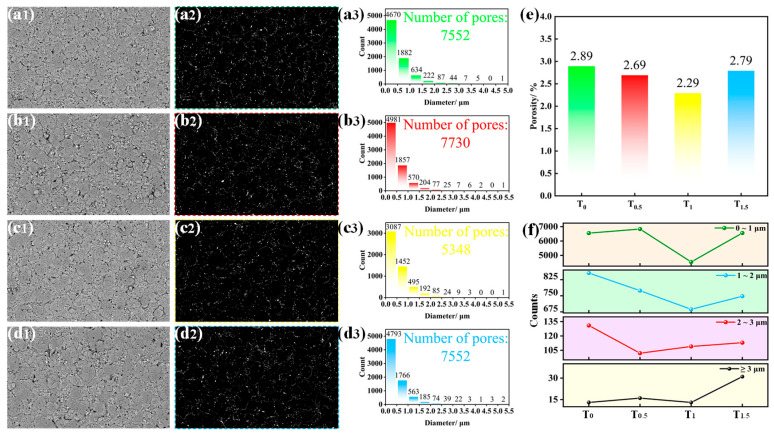
SEM surface morphologies and corresponding porosity data of MAO coatings: (**x1**; x = a, b, c, d) SEM surface morphologies of T_0_, T_0.5_, T_1_, and T_1.5_ coatings; (**x2**; x = a, b, c, d) Porosity measurement maps for T_0_, T_0.5_, T_1_, and T_1.5_ coatings; (**x3**; x = a, b, c, d) Pore size distribution maps for T_0_, T_0.5_, T_1_, and T_1.5_ coatings; (**e**) porosity values; (**f**) comparison of pore size distributions across different coatings.

Figure 3 presents the SEM surface morphologies and corresponding porosity data of micro-arc oxidation (MAO) coatings prepared with different amounts of doped TiO_2_ nanoparticles. As shown in Figure 3(x1) (where x = a, b, c, d), all MAO coatings exhibit uneven surfaces characterized by numerous crater-like pores. These pores are surrounded by pancake-like sintered regions, and the surfaces farther from the pores appear relatively rough, accompanied by a small number of micro-cracks. This characteristic morphology results from the following process: during the plasma arc discharge, numerous micropores known as “discharge channels” form. The high-temperature environment within these channels facilitates the rapid MAO reaction between aluminum and oxygen, producing Al_2_O_3_. Concurrently, the gas pressure and discharge pressure generated by the reaction increase sharply, ejecting molten Al_2_O_3_ from the discharge channels. This ejected molten Al_2_O_3_ is rapidly quenched and solidified upon contact with the surrounding electrolyte, eventually accumulating to form the “volcanic crater-like” protrusions.

**Figure 4:**
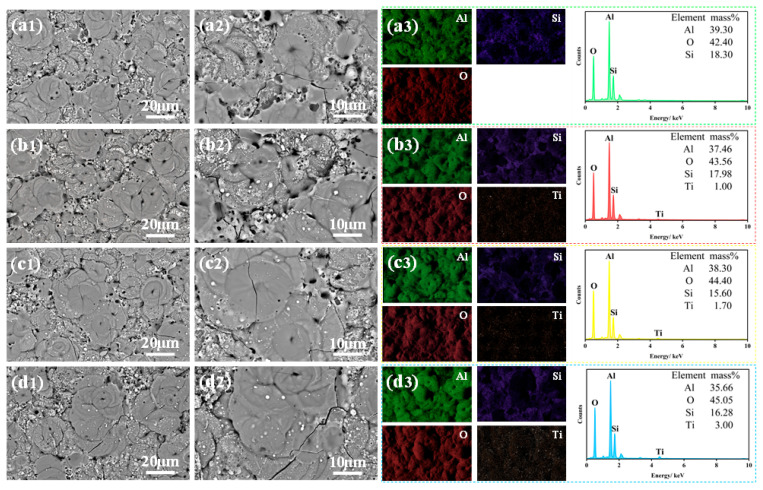
Scanning electron microscopy images and corresponding elemental distribution maps of the coatings: (**a1**–**a3**) T_0_; (**b1**–**b3**) T_0.5_; (**c1**–**c3**) T_1_; (**d1**–**d3**) T_1.5_.

To investigate the surface morphology of the coatings in greater detail, the surface porosity and pore size distribution were calculated and statistically analyzed. Figure 3(x2) (x = a, b, c, d) show the porosity measurement maps, Figure 4(x3) (x = a, b, c, d) illustrate the pore size distributions, and Figure 3e summarizes the surface porosity of the various MAO coatings. As observed in Figure 3(x3), the pore diameters predominantly fall within the range of 0–3 μm, with only a minority exceeding 3 μm. Furthermore, the number of pores decreases as their size increases. Figure 3e reveals that the surface porosity initially decreases and then increases with higher amounts of doped TiO_2_ nanoparticles. Specifically, the maximum and minimum porosity values are 2.98% (T_0_) and 2.29% (T_1_), respectively. The minimum porosity for the T_1_ coating is likely attributable to the partial filling of micropores and micro-cracks by an appropriate quantity of nanoparticles. Figure 3f compares the distribution of pores across different size ranges for each coating. The T1 coating demonstrates the lowest number of pores within the 0–1 μm, 1–2 μm, and >3 μm ranges, while also showing a relatively low count within the 2–3 μm range.

The addition of TiO_2_ nanoparticles to the electrolyte led to a reduction in the pore size of the MAO coatings. This reduction was particularly pronounced for the T_1_ coating, where TiO_2_ nanoparticles effectively filled some of the micropores and micro-cracks. However, with a further increase in TiO_2_ nanoparticle doping, the pore size gradually increased. This trend is likely due to the agglomeration and stacking of excessive TiO_2_ particles, resulting in prominent surface protrusions. This phenomenon can be explained as follows: with an appropriate amount of doped TiO_2_ nanoparticles, the localized high temperatures generated during the MAO process instantly melt a portion of the nanoparticles. Upon contact with the electrolyte, the molten TiO_2_ rapidly cools and solidifies, adhering to the gaps in the oxide coating. These solidified particles act as heterogeneous nucleation sites for the molten oxide, thereby reducing the size of the micropores in the surface’s porous layer. Furthermore, the size effect of the nanoparticles causes the electric field in the electrolyte to concentrate around them, making discharge channels more prone to form in the vicinity of the particles and resulting in higher discharge intensity. As the concentration of doped TiO_2_ nanoparticles increases, these intense and dispersed discharges generate a greater number of molten oxide regions. However, excessive TiO_2_ nanoparticles tend to agglomerate and stack upon embedding into the molten oxide, hindering its fluidity. This impediment prevents the molten material from adequately filling the pores, ultimately affecting the coating’s microstructure.

#### 2.3.2. EDS Analysis

Figure 4 shows the effect of TiO_2_ nanoparticle doping amount on the surface morphology and corresponding elemental distribution of the MAO coatings. The EDS mapping in Figure 4 reveals that after doping with TiO_2_ nanoparticles, the Ti element is uniformly distributed on the coating surface without significant agglomeration. This observation indicates that the amount of doped TiO_2_ nanoparticles has little influence on the uniformity of Ti element distribution. This phenomenon may be attributed to the random contact of TiO_2_ nanoparticles with the coating surface facilitated by the water-cooled circulation system. Concurrently, the electric arcs generated during the MAO process continuously move across the entire coating surface. This movement allows TiO_2_ nanoparticles to enter the plasma micro-arc zones through the discharge channels. Within these high-temperature zones, the nanoparticles undergo sintering and combine with the Al_2_O_3_ formed on the MAO surface to generate the ceramic layer.

Figure 4 also presents the contents of Al, O, Si, and Ti elements in the coatings with different TiO_2_ nanoparticle doping amounts. It can be observed that the Ti content in the coating gradually increases with a higher amount of doped TiO_2_ nanoparticles. In contrast, the influence of TiO_2_ nanoparticle doping on the Al, O, and Si element contents is relatively minor. The O content shows a slight overall increasing trend, while the Al and Si contents exhibit a slight decreasing trend. This phenomenon may be explained as follows: under the anodic current, the aluminum alloy substrate, acting as the anode, is oxidized to generate Al^3+^ ions. These ions migrate away from the substrate surface driven by the electric field force. As the doping amount of TiO_2_ nanoparticles increases, more TiO_2_ incorporates into the coating, leading to a gradual rise in Ti content. Simultaneously, the TiO_2_ nanoparticles may inhibit the contact between molten Al_2_O_3_ and Na_2_SiO_3_ in the electrolyte, thereby suppressing the incorporation of silicate species (${\mathrm{SiO}}_{3}^{2-}$) into the coating and resulting in a decrease in Si content.

### 2.4. Effect of TiO_2_ on Coating Cross-Sectional Morphology

**Figure 5:**
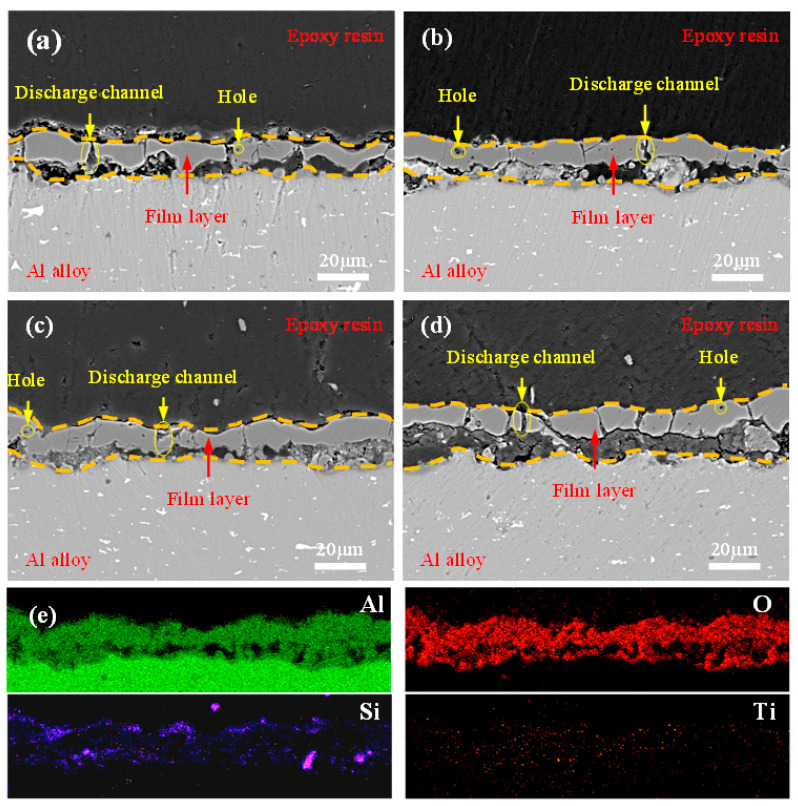
Cross-sectional SEM morphologies of MAO coatings and EDS elemental distribution of the T_1_ coating cross-section: (**a**) T_0_; (**b**) T_0.5_; (**c**) T_1_; (**d**) T_1.5_; (**e**) elemental distribution maps (Al, O, Si, Ti) for the T_1_ coating.

Figure 5 presents cross-sectional SEM morphologies of MAO coatings prepared with different amounts of doped TiO_2_ nanoparticles, along with EDS elemental mapping of the T_1_ coating cross-section. As shown in Figure 5, pores formed by arc discharges are present within all coatings. However, all coatings exhibit a tight bond with the substrate, demonstrating a typical metallurgical bonding interface. The coating thickness gradually increases with a higher amount of doped TiO_2_ nanoparticles, which is consistent with the results from Figure 2a. Specifically, the T_0_ coating contains numerous discharge channels. Although the coating is continuous, it is not sufficiently dense, as visible in Figure 5a. With an increased doping amount (T_0.5_), the coating thickness grows further. While some fine pores appear within the coating, its continuity and density are significantly improved, as seen in Figure 5b. The continuity and density of the T_1_ coating are further enhanced, with only a few discharge channels remaining within its structure, as shown in Figure 5c. However, for the T_1.5_ coating, despite a further increase in thickness, the discharge channels become noticeably larger, and the internal pores also increase in size, as evident in Figure 5d. Among all samples, the T_1_ coating exhibits the most optimized internal structure. This structural evolution can be explained as follows: doping the electrolyte with an appropriate amount of TiO_2_ nanoparticles increases its electrical resistance, leading to a higher electric field intensity. This, in turn, promotes more uniform and intensive arc discharges. The enhanced discharges facilitate the generation of a greater volume of molten material, which deposits on the coating surface, thereby filling and repairing defects such as micropores and microcracks, ultimately improving coating quality. Conversely, when the doping amount exceeds a critical value, excessive nanoparticles tend to agglomerate during their incorporation into the molten oxide. This agglomeration hinders the fluidity of the molten material, making it difficult to fill the pores effectively.

To investigate the distribution of TiO_2_ nanoparticles within the coating, cross-sectional EDS analysis was performed on the T_1_ coating, with results presented in Figure 5e. The analysis indicates that Al, O, Si, and Ti elements are uniformly distributed across the coating cross-section, which is attributed to the dense microstructure of the coating. This uniform elemental distribution provides robust structural support for the formation of discharge channels.

### 2.5. Effect of TiO_2_ on Phase Composition of the Coatings

**Figure 6:**
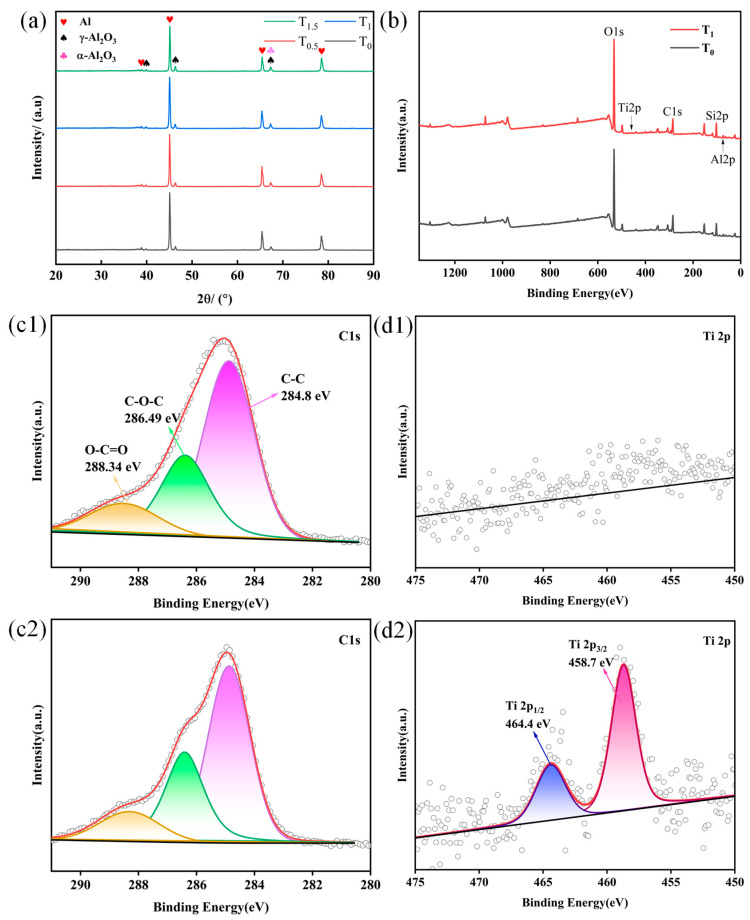
(**a**) XRD patterns of the MAO coatings; (**b**) XPS survey spectra of the T_0_ and T_1_ coatings; (**c1**,**c2**) high-resolution C 1 s spectra of the T_0_ and T_1_ coatings; (**d1**,**d2**) high-resolution Ti 2p spectra of the T_0_ and T_1_ coatings.

Figure 6 presents the XRD patterns of MAO coatings prepared with different amounts of doped TiO_2_ nanoparticles, along with the XPS survey spectra and high-resolution C 1 s and Ti 2p spectra for the T_0_ and T_1_ coatings. As shown in Figure 6a, the MAO coatings are primarily composed of α-Al_2_O_3_ and γ-Al_2_O_3_ phases, regardless of the TiO_2_ nanoparticle concentration. With increasing TiO_2_ doping, the intensity of the diffraction peaks corresponding to the aluminum substrate gradually decreases. In contrast, the intensities of the peaks for the α- Al_2_O_3_ and γ- Al_2_O_3_ phases show an opposite trend relative to the Al substrate peak. Overall, the variation in the intensity of these characteristic peaks with increasing doping amount is relatively minor. No distinct TiO_2_ phase was detected in the XRD patterns. The highly uniform dispersion of the as-fabricated TiO_2_ nanoparticles (5–10 nm) within the composite coating contributes to the absence of distinct TiO_2_ crystalline phase peaks in the XRD patterns under the present experimental conditions. This can be attributed to two interrelated factors. First, the crystallite size approaches the lower detection limit of conventional XRD for nanocrystalline phases. Second, the low concentration and highly dispersed state of the nanoparticles within the coating further attenuate the cumulative intensity of the characteristic diffraction signals.

To verify the presence of Ti, XPS analysis was performed. The XPS survey spectra in Figure 6b confirm the presence of Al, O, Si, and Ti elements, which is consistent with the EDS results (Figure 4). The detected C element is attributed to atmospheric contamination. The high-resolution C 1 s spectra for both T_0_ and T_1_ coatings Figure 6(c1,c2) can be deconvoluted into three peaks at binding energies of 284.8 eV, 286.49 eV, and 288.34 eV. These are assigned to C-C, C-O-C, and O-C=O bonds, respectively [28]. Figure 6(d1,d2) show the high-resolution Ti 2p XPS spectra for the T_0_ and T1 coatings, respectively. The spectrum for the T_1_ coating, which contains doped TiO_2_, displays two characteristic peaks at binding energies of 458.7 eV and 464.4 eV. These correspond to the Ti 2p_3/2_ and Ti 2p_1/2_ orbitals, respectively, with a spin–orbit splitting energy of 5.7 eV [29]. The presence of these Ti signals suggests the successful incorporation of TiO_2_ nanoparticles into the MAO coating.

### 2.6. Effect of TiO_2_ on the Corrosion Resistance of the Coatings

**Figure 7:**
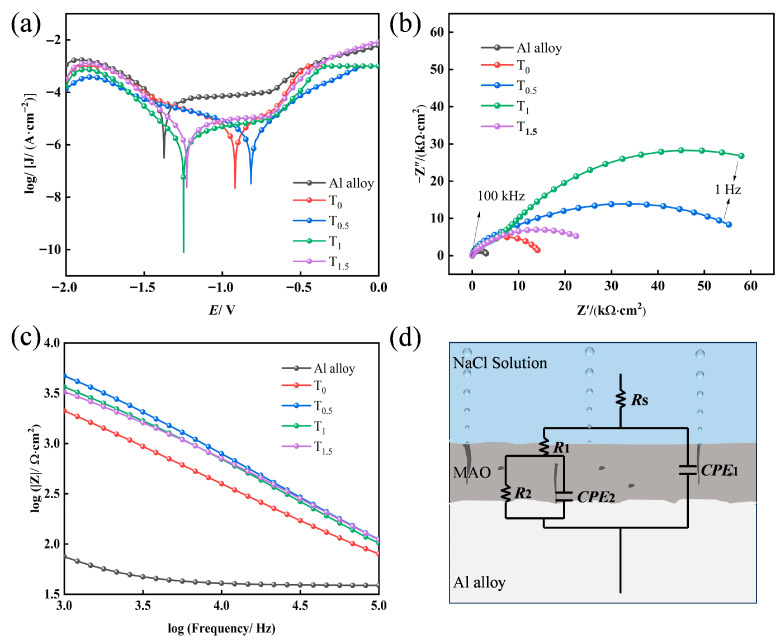
Electrochemical measurements of MAO coatings in 3.5 wt% NaCl solution: (**a**) potentiodynamic polarization curves; (**b**) Nyquist plots; (**c**) equivalent circuit model for EIS data fitting; (**d**) Fitted equivalent circuit model.

Figure 7 shows the potentiodynamic polarization curves and electrochemical impedance spectroscopy (EIS) with the corresponding equivalent circuit model for MAO coatings prepared with different amounts of doped TiO_2_ nanoparticles, tested in a 3.5 wt% NaCl solution. The electrochemical parameters derived from fitting the polarization curves are listed in Table 1. In MAO coatings, *E*_corr_ may fluctuate due to surface conditions (e.g., porosity, phase composition). However, a significant decrease in *J*_corr_ coupled with a substantial increase in *R*_p_ indicates a substantial improvement in corrosion resistance. Tafel extrapolation analysis yielded corrosion current densities (*J*_corr_) of 2.911 × 10^−6^, 1.835 × 10^−6^, 1.127 × 10^−6^, and 2.744 × 10^−6^ A·cm^−2^ for the T_0_, T_0.5_, T_1_, and T_1.5_ coatings, respectively. In comparison, the bare substrate exhibited a significantly higher *J*_corr_ of 2.446 × 10^−5^ A·cm^−2^. These results suggest that the MAO treatment significantly enhances corrosion resistance. Notably, the T_1_ coating demonstrated the best performance, with its polarization resistance (*R*_p_) showing an approximately 130% increase compared to the T_0_ coating, indicating that an optimal doping amount further enhances corrosion resistance.

To gain deeper insight into the effect of TiO_2_ nanoparticle doping on corrosion behavior, EIS analysis was conducted. The Nyquist plots for the different coatings are presented in Figure 7b, The local high-frequency Bode plots for the different coatings are presented in Figure 7c, and the fitted equivalent circuit model is shown in Figure 7d. The corresponding fitting parameters are summarized in Table 2. The equivalent circuit consists of: *R*_s_ (the resistance of the 3.5% NaCl solution), CPE_1_(Y_1_, n_1_) and R_1_ (the constant phase element and resistance associated with the porous outer layer of the MAO coating), and CPE_2_(Y_2_, n_2_) and *R*_2_ (the constant phase element and charge transfer resistance associated with the dense inner layer/substrate interface). The corrosion resistance of the samples in the solution can be qualitatively assessed by the diameter of the capacitive loop in the Nyquist plot. A larger loop radius corresponds to a lower corrosion rate and a stronger barrier capability against corrosive media. As seen in Figure 7b, the diameter of the capacitive loop initially increases and then decreases with higher TiO_2_ nanoparticle doping, with the T_1_ coating exhibiting the largest radius. This suggests that the T_1_ coating possesses the best corrosion resistance among the series. As can be seen from Figure 7c, although the impedance modulus (log∣Z∣) of T_0.5_ is slightly higher than that of T_1_ in the high-frequency region, the arc radius of T_1_ in the low-frequency Nyquist plot exceeds that of T_0.5_. Given that low-frequency impedance primarily reflects charge transfer resistance, which is directly related to the long-term barrier capability of the coating against the penetration of corrosive media, the superiority of T_1_ in the low-frequency range indicates its enhanced long-term protective performance. This allows for more effective inhibition of corrosion reactions. The corrosion resistance of the coatings is fundamentally governed by the protective quality of the dense inner layer, which is reflected by the resistance *R*_2_. A higher *R*_2_ value indicates a greater ability of the coating to resist the penetration of corrosive electrolytes. As listed in Table 2, the T_1_ sample exhibits the highest *R*_2_ value, signifying that it presents the greatest resistance to the ingress of corrosive ions, thereby offering superior corrosion protection. This conclusion is consistent with the results obtained from the polarization curve analysis.

**Figure 8:**
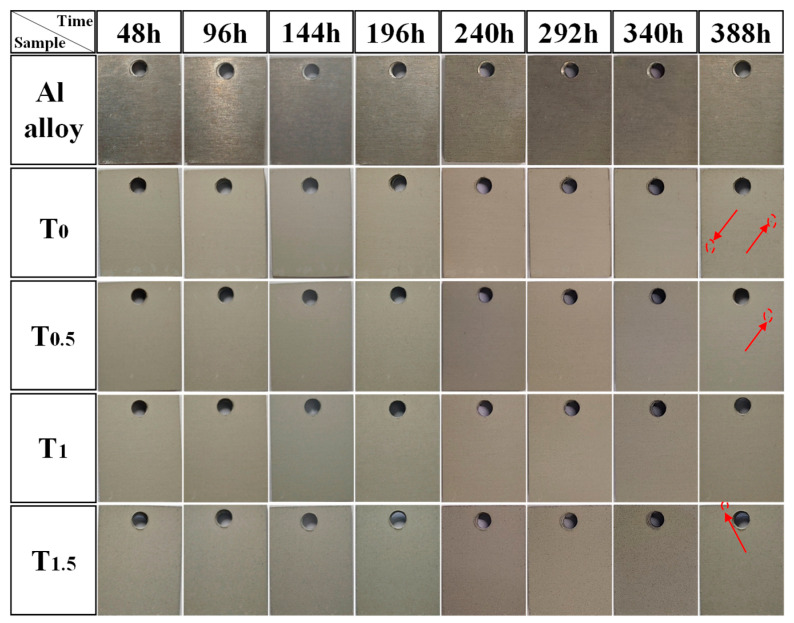
Macroscopic surface morphologies of the samples after immersion.

To further investigate the corrosion resistance of the MAO coatings, immersion tests were conducted by exposing different samples to a 3.5 wt% NaCl solution. The macroscopic surface morphologies of the samples after immersion are shown in Figure 8. As observed in Figure 8, after 144 h of immersion, the metallic luster of the bare 6061 aluminum alloy completely disappeared, and its surface exhibited dull, slight corrosion marks. The T_0_, T_0.5_, and T_1.5_ samples showed minor pitting corrosion after 388 h. Throughout the entire immersion test, no obvious corrosion pits were observed on the T_1_ coating. These results indicate that the T_1_ coating possesses the best corrosion resistance among all tested samples. This superior performance is attributed to the low porosity and high density of the T_1_ coating, which together effectively enhance its barrier properties against corrosive media.

**Figure 9:**
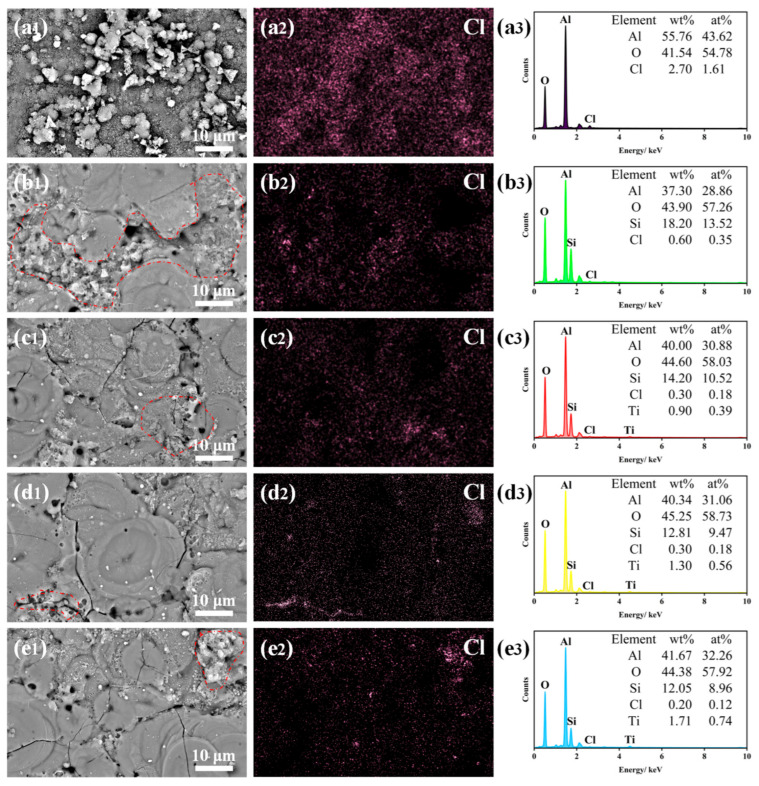
SEM images, corresponding Cl element distribution maps, and EDS spectra of the coatings after immersion test: (**a1**–**a3**) bare aluminum alloy; (**b1**–**b3**) T_0_; (**c1**–**c3**) T_0.5_; (**d1**–**d3**) T_1_; (**e1**–**e3**) T_1.5_.

Further observation of the microscopic morphologies Figure 9(x1), where x = a, b, c, d, e reveals that, after the same immersion duration, the amount of corrosion products precipitated on the coating surfaces shows a trend of initial decrease followed by stabilization. EDS analysis of the immersed coatings Figure 9(x2,x3), x = a, b, c, d, e indicates the presence of Cl element, most likely in the form of Cl^−^ ions. As the doping amount of TiO_2_ nanoparticles increases, the detected Ti content rises, while the Cl content decreases. This suggests that the enrichment of TiO_2_ nanoparticles and the subsequent formation of corrosion-related products may provide short-term protective effects, thereby enhancing the coating’s resistance to corrosive media. Among the samples, the T_1_ coating exhibits the least amount of precipitated corrosion products on its surface, allowing it to be identified as possessing the optimal corrosion resistance. This finding is consistent with the conclusions drawn from the earlier potentiodynamic polarization tests.

**Figure 10:**
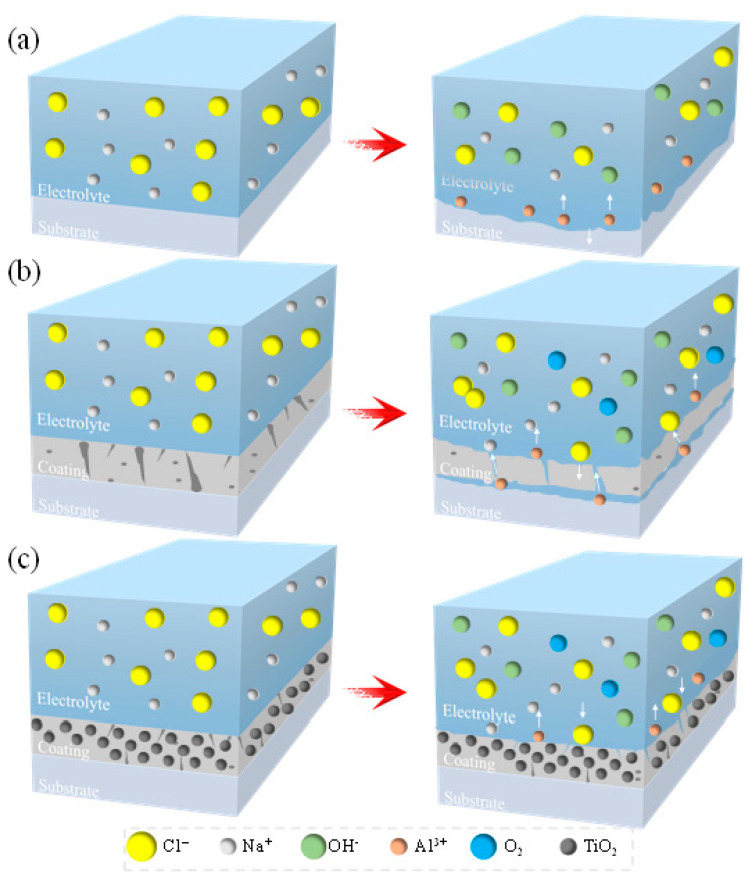
Schematic of corrosion resistance mechanisms: (**a**) Bare substrate; (**b**) MAO coating; (**c**) MAO coating modified with TiO_2_ nanoparticles.

Figure 10 schematically illustrates the corrosion protection mechanisms of the bare substrate, the unmodified MAO coating, and the TiO_2_ nanoparticle-modified MAO coatings. When exposed to a NaCl solution, corrosive ions must penetrate the coating through defects such as micropores and micro-cracks to reach and attack the substrate. As depicted in Figure 10a, the unprotected substrate surface is in direct contact with the NaCl solution, allowing corrosive ions to react rapidly with the substrate and leading to swift corrosion. Figure 10b represents the case of an MAO-coated sample. The corrosive medium from the NaCl solution must first infiltrate through surface discharge channels and micro-cracks in the MAO coating before reaching the substrate. Over extended immersion, corrosion products gradually accumulate on the coating surface.

The incorporation of an optimal amount of TiO_2_ nanoparticles into the electrolyte modifies this process in two key ways. First, it increases the electrical resistance of the electrolyte system, leading to a higher electric field intensity. This promotes more uniform and intensive arc discharges, which in turn facilitates the generation and deposition of a greater volume of molten material. This material effectively fills and repairs defects like micropores and cracks within the coating, thereby improving its overall quality and density. Second, the TiO_2_ nanoparticles dispersed within the MAO coating alter the penetration pathways for corrosive media, enhancing the coating’s barrier effect. Corrosive agents are forced to navigate more tortuous paths to permeate the coating. Consequently, as shown in Figure 10c, the coating doped with an optimal amount of TiO_2_ nanoparticles can more effectively hinder the penetration of corrosive ions, significantly boosting its corrosion resistance. However, with a further increase in TiO_2_ nanoparticle doping (e.g., in the T_1.5_ coating), the number of discharge channels increases, and the coating’s compactness decreases. This results not only in a greater number of penetration pathways for corrosive ions but also in larger pore sizes, which collectively exert a significant detrimental effect on the coating’s corrosion resistance.

It should be noted that the discussion concerning the influence of TiO_2_ nanoparticles on discharge behavior and coating growth kinetics during micro-arc oxidation in this study is primarily based on a correlative analysis of macroscopic coating properties (e.g., thickness, roughness), microstructure (characterized by SEM and XRD), and electrical signal waveforms (voltage-time curves). While the results strongly suggest that the nanoparticles affect discharge intensity, distribution, and coating composition—likely through mechanisms such as altering electrolyte conductivity, participating in reactions within discharge channels, and becoming physically embedded—the precise physicochemical processes involved remain inferred from these indirect observations. Specifically, how nanoparticles modulate plasma characteristics within individual discharge channels or alter the dielectric breakdown threshold cannot yet be directly resolved due to the current experimental limitations in performing in situ observation of single discharge events at micro- to nanosecond timescales. This represents an acknowledged constraint in the present work and indicates a direction for future investigation.

It should be noted that, in addition to further elucidating the aforementioned microscopic mechanisms, the long-term electrochemical stability of the coatings and the evolution of their impedance over time are also critical indicators for assessing their potential in engineering applications. This will constitute another important focus for our future research.

## 3. Materials and Methods

### 3.1. Materials

The substrate was extruded 6061 aluminum alloy; its chemical composition is listed in Table 3. Samples with dimensions of 20 mm × 15 mm × 3 mm were cut by wire electrical discharge machining. Prior to MAO, the surfaces were ground sequentially with 400–1200 grit SiC papers and then polished to remove the native oxide and ensure smoothness. The specimens were ultrasonically cleaned in deionized water for 5 min and dried for later use.

### 3.2. Electrolyte and Nanoparticles

The electrolyte for MAO comprised 16 g·L^−1^ Na_2_SiO_3_·9H_2_O, 3 g·L^−1^ NaOH, 2 g·L^−1^ KF, and 3 g·L^−1^ C_3_H_8_O_3_ (all reagents from Macklin). To investigate the influence of TiO_2_ nanoparticles (from Macklin (Shanghai, China), with a purity of ≥99.8%, an average particle size of approximately 5–10 nm, an anatase crystal structure, and amphiphilic surface properties) on the MAO coatings, TiO_2_ was added at 0, 0.5, 1.0, and 1.5 g·L^−1^, designated as T_0_, T_0.5_, T_1_, and T_1.5_, respectively. For electrolyte preparation, TiO nanoparticles were first dispersed into 1 L of electrolyte by ultrasonication for 30 min to ensure uniform dispersion, then transferred to the MAO tank under continuous stirring to maintain homogeneity.

### 3.3. MAO Setup

**Figure 11:**
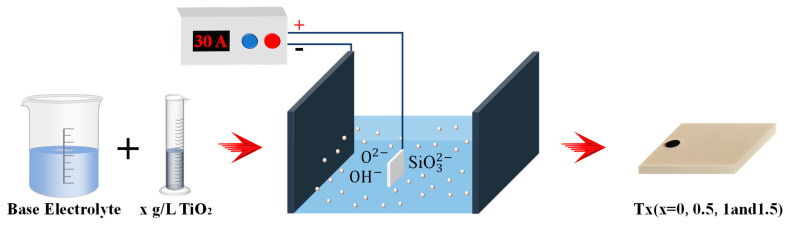
A schematic of the MAO setup.

Figure 11 shows a schematic of the MAO setup, which consisted of a 720 V/30 A power supply, a U-shaped stainless-steel cathode, an electrolytic tank, and a water-cooling circulation system. During processing, the 6061 alloy specimen served as the anode and the U-shaped stain-less-steel plate as the cathode. The electrolyte temperature was controlled at 20 ± 2 °C. The MAO parameters were oxidation time 20 min, duty cycle 30%, frequency 1000 Hz, and current density 6 A·dm^−2^.

### 3.4. Structural Characterization of the Coatings

A Minitest 2500 eddy-current thickness gauge (ElektroPhysik, Cologne, Germany) was used to measure coating thickness at 25 random points on both sides of each sample; outliers were removed and the average was reported. Surface roughness was measured with a JITAI820 profilometer (Beijing JITAI Inspection Equipment Co., Ltd., Beijing, China) at five random points on each side, and the average value was taken. SEM (Phenom XL G2, Funano Scientific Instruments (Shanghai) Co., Ltd., Shanghai, China) was employed to examine surface and cross-sectional morphologies, and EDS was used to determine surface elemental compositions. Three different regions of each sample were randomly selected for SEM imaging, with each image covering an area of approximately 0.11 mm^2^. Image analysis was performed using ImageJ software (version 1.54m): the original SEM images were first converted to 16-bit grayscale, followed by binarization segmentation using the ‘Auto Threshold’ function to distinguish pores from the substrate. The threshold processing method was kept consistent across all samples to ensure comparability of the analytical results. XRD (DX-2700B) (Dandong Hao Yuan Instrument Co., Ltd., Dandong, China) was used to identify phases with a scan speed of 10°·min^−1^ over 2θ = 20–90°. XPS (Thermo Scientific K-Alpha) (Thermo Fisher Scientific Inc., Waltham, MA, USA) was used to analyze chemical states; binding energies were calibrated to the adventitious C 1 s peak at 284.8 eV.

### 3.5. Corrosion Characterization

Electrochemical tests were conducted on a CHI660E workstation (Shanghai Chenhua Instrument Co., Ltd., Shanghai, China) using a standard three-electrode cell with the sample as working electrode, a saturated calomel electrode as reference, and a platinum counter electrode in 3.5 wt% NaCl solution.

Prior to electrochemical testing, all specimens were immersed in a 3.5 wt% NaCl solution for 30 min to attain a stable open-circuit potential. The polarization curves were measured at a scan rate of 5 mV/s over a potential range from −2 V to 0 V. During the preparation of the working electrode, only a 0.785 cm^2^ coated surface was exposed as the test area to ensure a consistent exposed area for each measurement. The electrochemical impedance spectroscopy (EIS) measurements were conducted under the following parameters: a frequency range of 10^5^ Hz to 10^−2^ Hz, with 12 points collected per decade, and an excitation voltage amplitude of 10 mV. To ensure statistical validity, at least three parallel samples were prepared for each treatment condition, and each sample underwent complete EIS and potentiodynamic polarization curve testing. The spectra and data points presented in the manuscript represent typical results from no fewer than three independent tests.

The test data were analyzed using ZView2 software (version 2.1c), and the EIS data were fitted based on an equivalent circuit model. The goodness of fit was evaluated through the chi-square value (χ^2^) and the fitting error of each circuit element. In this study, the χ^2^ values for the fitting results were typically below 10^−3^, and the fitting errors for the parameters of each component were generally less than 10%, indicating good fitting quality and that the model effectively describes the interfacial processes.

The long-term immersion experiments in this study were conducted at room temperature, with the solution temperature consistently maintained at 25 ± 1 °C. Throughout the immersion period, the 3.5 wt% NaCl solution was renewed every 48 h. Prior to each renewal, the solution pH was measured and confirmed to remain stable within the range of 6.5 ± 0.3. No active pH adjustment was performed, in order to simulate a natural corrosion environment. For each experimental condition, three parallel samples were prepared.

## 4. Conclusions

This study systematically investigated the effects of doping with TiO_2_ nanoparticles (samples T_0_, T_0.5_, T_1_, and T_1.5_) on the microstructure, composition, and properties of micro-arc oxidation (MAO) coatings on 6061 aluminum alloy. The main conclusions are as follows:

1. TiO_2_ nanoparticles were uniformly distributed within the MAO coating. The primary phase composition consisted of α-Al_2_O_3_ and γ-Al_2_O_3_. While doping had minimal influence on the phase composition, it significantly altered the coating’s microstructure, leading to increased surface roughness and thickness with higher doping levels.
2. Appropriate TiO_2_ doping reduced the coating porosity and improved its density, thereby enhancing corrosion resistance. However, for the sample with the highest addition amount studied (T_1.5_), a decrease in coating densification was observed compared to the optimally doped T_1_ sample, resulting in diminished corrosion performance.
3. Within the experimental scope, the T_1_ coating exhibited the optimal corrosion resistance. This study provides insights for optimizing MAO processes and designing protective coatings for 6061 aluminum alloys in chloride-containing environments. It demonstrates that controlling the doping amount of TiO_2_ nanoparticles is a critical factor for improving the corrosion resistance of MAO coatings.

## Figures and Tables

**Table 1:** Electrochemical parameters obtained from fitting the polarization curves.

	*E*_corr_/V	*J*_corr_/(A·cm^−2^)	*R*_p_/(Ω·cm^2^)
Al alloy	−1.374	2.446 × 10^−5^	1.964 × 10^3^
T_0_	−0.919	2.911 × 10^−6^	1.538 × 10^4^
T_0.5_	−0.817	1.835 × 10^−6^	2.245 × 10^4^
T_1_	−1.248	1.127 × 10^−6^	3.558 × 10^4^
T_1.5_	−1.228	2.744 × 10^−6^	1.433 × 10^4^

**Table 2:** Equivalent circuit parameters.

	*R*s/(Ω·cm^2^)	Y_1_/ (Ω^−1^·s^n^·cm^−2^)	*n*_1_	*R*_1_/(Ω·cm^2^)	Y_2_/ (Ω^−1^·s^n^·cm^−2^)	*n*_2_	*R*_2_/(Ω·cm^2^)	Equivalent Model
6061	38.55	8.145 × 10^−6^	0.877	3.204 × 10^3^	----	----	----	R(QR)
T_0_	35.43	5.108 × 10^−8^	0.919	2.782 × 10^3^	5.927 × 10^−7^	0.659	1.220 × 10^4^	R{Q[R(QR)]}
T_0.5_	26.98	6.229 × 10^−8^	0.895	7.303 × 10^3^	2.683 × 10^−6^	0.506	5.889 × 10^4^	R{Q[R(QR)]}
T_1_	28.30	4.720 × 10^−8^	0.777	9.538 × 10^3^	1.664 × 10^−6^	0.756	7.859 × 10^4^	R{Q[R(QR)]}
T_1.5_	33.29	4.621 × 10^−8^	0.919	1.407 × 10^3^	2.375 × 10^−6^	0.522	2.826 × 10^4^	R{Q[R(QR)]}

**Table 3:** Chemical composition of 6061 aluminum alloy (wt%).

Si	Mg	Fe	Cu	Mn	Cr	Zn	Ti	Al
0.4~0.8	0.8~1.2	0.7	0.15~0.4	0.15	0.25	0.25	0.15	Bal

## Data Availability

The data are contained within the article.
